# A Transflective Nano-Wire Grid Polarizer Based Fiber-Optic Sensor

**DOI:** 10.3390/s110302488

**Published:** 2011-02-28

**Authors:** Jing Feng, Yun Zhao, Xiao-Wen Lin, Wei Hu, Fei Xu, Yan-Qing Lu

**Affiliations:** College of Engineering and Applied Sciences and National Laboratory of Solid State Microstructures, Nanjing University, Nanjing 210093, China; E-Mails: feng.jing.nju@gmail.com (J.F.); njuzhaoyun@gmail.com (Y.Z.); bonbon_grace@163.com (X.-W.L.); huwei@nju.edu.cn (W.H.); feixu@nju.edu.cn (F.X.)

**Keywords:** subwavelength structures, fiber optics sensors, pressure measurement, polarization-selective devices

## Abstract

A transflective nano-wire grid polarizer is fabricated on a single mode fiber tip by focused ion beam machining. In contrast to conventional absorptive in-line polarizers, the wire grids reflect TE-mode, while transmitting TM-mode light so that no light power is discarded. A reflection contrast of 13.7 dB and a transmission contrast of 4.9 dB are achieved in the 1,550 nm telecom band using a 200-nm wire grid fiber polarizer. With the help of an optic circulator, the polarization states of both the transmissive and reflective lights in the fiber may be monitored simultaneously. A kind of robust fiber optic sensor is thus proposed that could withstand light power variations. To verify the idea, a fiber pressure sensor with the sensitivity of 0.24 rad/N is demonstrated. The corresponding stress-optic coefficient of the fiber is measured. In addition to pressure sensing, this technology could be applied in detecting any polarization state change induced by magnetic fields, electric currents and so on.

## 1. Introduction

In the past two decades, fiber-optic sensors have attracted increasing attention in both the academic and industrial communities. In comparison with electrical sensors, fiber-optic technology has many advantages, such as electromagnetic immunity, miniature size, light weight, passive composition, high temperature compatibility and multiplexing capability. As a result, a variety of fiber-optic sensing applications have been proposed to measure various physical parameters such as pressure, temperature, acceleration, voltage and current. In addition, the fast-growing optical fiber communication industry has resulted in a substantial reduction in optical fiber sensor cost. It is even possible to integrate sensing and communication functions together to enable future applications in “smart grids” and “the internet of things” [1,2].

On the other hand, there have been many studies on metamaterials in the past 10 years. A metamaterial is a macroscopic composite of periodic or non-periodic structure, whose function is due to both the cellular architecture and the chemical composition [3]. The dependence of metamaterial properties on the architecture provides great flexibility to control metamaterials. Waveguided or planar metamaterials which are composed of complimentary structures like nano-wire grids have been used to design optical components like filters, beam splitters, couplers, polarizers and interferometers [4–6]. It would be interesting to apply these structures in optical fiber sensing buildups [7,8].

In this work, a nano-wire grid fiber polarizer (NWGFP), which is a gold grid array fabricated on a fiber tip by a focused ion beam, was developed for sensing applications. In contrast to conventional absorptive polarizers, the incident TE-mode light is reflected by the metal wire grid, while TM-mode light could pass through with a low loss. Different polarized lights in the fiber thus experience different transmittance and reflectance. A 13.7 dB reflection contrast and a 4.9 dB transmission contrast are experimentally obtained between TE and TM modes, which is sensitive enough to monitor tiny polarization changes in a fiber. A photoelastic pressure sensor with the sensitivity of 0.24 rad/N was thus obtained. Because of the transflective feature of NWGFP, the polarization states of both the transmissive and reflective lights in the fiber may be monitored simultaneously. The fiber optic sensor thus can withstand light power variations showing great stability. Further extensions of our NWGFP technique are also discussed.

## 2. Nano-Wire Grid Polarizer

Spillman reported a photoelastic fiber-optic pressure sensor for the first time in 1982 [9]. This kind of pressure sensing technique is based on instant detection of polarization changes resulting from photoelastic effects. A high-contrast polarizer is required for pressure sensing purposes. Obviously a fiber in-line polarizer is desirable for practical applications due to its low loss and simple package. Although various approaches have been studied [10–12], in this paper we propose to fabricate a fiber polarizer making use of the characteristics of nano-wire grids.

**Figure 1.**
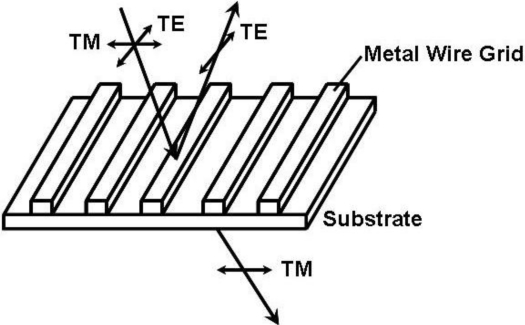
The working principle of a wire grid polarizer.

Figure 1 shows the general working principle of a wire grid polarizer (WGP). The grids should be subwavelength metallic structures, which means that the period is far smaller than the incident wavelength. Satisfying this condition, the input TE-mode light (electric vector parallel to the grids) is reflected by the metal grids, while the TM-mode light (electric vector perpendicular to the grids) is transmitted. Although the WGP was originally demonstrated in the radio frequency more than 100 years ago [13], it has been successfully extended to the near infrared and visible region [14,15] due to the fast growing micro-fabrication techniques.

## 3. Experiments and Discussion

**Figure 2.**
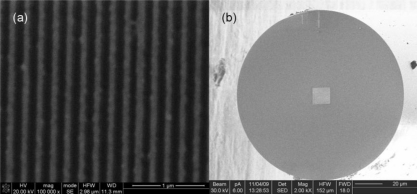
**(a)** SEM image of part of the gold grid array fabricated on a fiber tip; **(b)** SEM image of a whole fiber tip with a gold grid array at its core area.

Similar to bulk WGPs, we intended to fabricate metal grids on fiber tips, which theoretically would result in an in-line fiber polarizer. We choose gold as the grid material, due to its stability and good polarizing performance [16]. A fiber is cut into 3 cm-long segments, both with two flat end faces. Then a 70 nm-thick gold film is deposited onto one end face of the fiber segment by ion beam sputtering. A grid array (with the size of 12 μm × 12 μm) is made in the film with a focused-ion-beam machining system (Strata FIB 201, FEI company, 30 keV Ga ions). In our experiment, an SMF-28 compatible fiber is employed, whose mode field diameter at 1,550 nm is 10.4 ± 0.8 μm. Therefore the machined nanostructured area is large enough to cover the fiber mode and modulate the light. What’s more, to ensure a good polarizing performance at 1,550 nm, the period of the gold grid is designed at 200 nm, far smaller than the light’s wavelength. Figure 2(a) displays the SEM image of the gold grid array we fabricated on a fiber end face, whose period is 202 nm and duty cycle is 51%. From Figure 2(b), the array is selectively machined that covers the core area of the fiber. It takes only several minutes to accomplish fabrication. Once the fiber with gold grid is further connected with two normal fibers at its two ends, a compact NWGFP is thus obtained.

**Figure 3.**
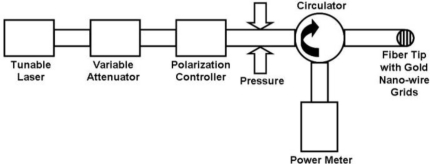
Experimental setup to demonstrate the NWGFP and pressure sensor.

Figure 3 depicts the schematic diagram of our experimental setup to characterize the NWGFP and demonstrate its sensing capability. The 1550 nm light from a tunable laser (Santec TSL-210) enters a variable attenuator (EXFO 3100). Then we may further adjust the input light through a polarization controller to realize crossed or parallel polarization interference. After the polarization controller, the light goes into a circulator through its “input” port. The NWGFP is connected to its “output” port. The reflected light is measured by a fiber power meter (HP 8153A), which is connected to the circulator through its “monitor” port. Meanwhile, the transmitted light is measured simultaneously with a lightwave multimeter (Agilent 8,163B).

The NWGFP reflects TE-mode light and transmits TM-mode light. If the input light is in TE mode, it will be reflected and merely transmitted. In this circumstance, the whole setup is like two parallel polarizers for reflection, while this configuration works like a typical cross-polarization interference filter from transmission point of view. If the input light is in TM mode, the situation is just the opposite. Any phase-retardation induced in the fiber between the polarization controller and our NWGFP can be detected according to the output power variation, which is the fundamental of our photoelastic pressure sensor.

Before the pressure measurement, the NWGFP is characterized. The reflection contrast ratio of the NWGFP is measured to be 13.7 dB, and the transmission contrast ratio is 4.9 dB. The contrast is still not high enough to meet the requirement of commercial fiber polarizers. In our opinion, we can improve NWGFP’s polarizing performance by ameliorating the gold film quality and choosing more suitable values of grid parameters like depth, duty cycle, period and so on, and adopting more precise focused-ion-beam machining system or nano-imprint technology are also believed able to improve its performance.

Although our NWGFP is still not ideal, it is already adequate for pressure sensing. A 368 cm-long fiber between the polarization controller and circulator is wound to form a coil and placed between two 15 cm × 15 cm flat glass plates on an optical table. This coil has direct contact with the top and bottom glass plates. Then balance weights with calibrated mass are applied to the top plate gradually, resulting in an increased pressure to the fiber core area uniformly. Because the applied forces are transferred to the fiber’s axial cross section, whose area is *2lr* (*l* is the length of the fiber coil, and *r* is the fiber radius), the pressure at the fiber core thus could be calculated from the corresponding force and area:

(1) P=Mg/2lr

where *P* is the pressure applied to the fiber core, *M* is the mass of the balance weights applied.

In our pressure sensing experiment, the polarization controller is set in the way that the reflection reaches the minimum, to obtain a “dark” state with cross-polarizers. As discussed before, the applied pressure results in birefringence in the fiber due to the photoelastic effect. There is thus phase retardation between the TM- and TE-mode lights. Once there is a little change of the light mode inside the fiber, there would be a detectable variation of the output light intensity. We can use Mueller matrices [17,18] to clarify this optical process:

(2) $$I_{\mathrm{ref}}=\frac{I_{0}}{2}{\text{sin}}^{2}\frac{\delta }{2}$$

(3) $$I_{\mathrm{trans}}=\frac{I_{0}}{2}{\text{cos}}^{2}\frac{\delta }{2}$$

where *I_ref_* and *I_trans_* are the light intensities of the reflection and transmission output, *I_0_* is input light intensity, and *δ* is phase retardation.

**Figure 4.**
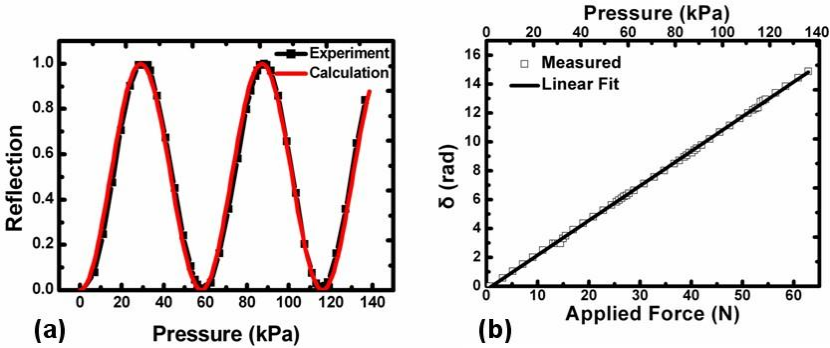
**(a)** The pressure induced reflection change. The red curve is calculated based on the stress-optic coefficient acquired from the experiment result; **(b)** The measured phase retardation as a function of the applied force. The solid line is a best-fit curve.

At the beginning, the variable attenuator is fixed and we only monitor the reflection output change. When no pressure is applied, almost no output light is detected as it cannot pass a pair of orthogonally-placed cross-polarizers. When the pressure becomes higher, the output intensity goes up and down forming a typical sinusoidal curve, as shown by the black dotted curve in Figure 4(a). As a consequence, the pressure induced phase retardation can be easily deduced from Equation (2). The result is shown in Figure 4(b) with x-axis title of the applied force *F*. Thus, the sensitivity of this pressure sensor is calculated to be *dδ/dF* = 0.24 rad/N.

Assuming the fiber is fully elastic and mechanically homogeneous, the photoelastic phase retardation induced by the applied force is given by [19]:

(4) δ=8CF/λr

where *C* is the stress-optic coefficient, and *λ* is the light wavelength. So, a fiber should have the pressure sensitivity of:

(5) dδ/dF=8C/λr

According to Equation (5), the fiber’s stress-optic coefficient, light wavelength and fiber diameter all play important roles in the sensitivity of such a pressure sensor. Given our measured sensitivity 0.24 rad/N, *C* is calculated to be 3 × 10^−12^ m^2^/N. To our knowledge, there are few reports on the stress-optic coefficient measurement of fused silica fiber in the 1,550 nm telecomm band. However, this result is consistent with the tendency of measured visible band values [20,21]. It is also similar to a result 3.03 × 10^−12^ m^2^/N we previously measured [22], and another extrapolated result of about 3.1 × 10^−11^ m^2^·kg^−1^ at 1,550 nm reported by Barlow and Payne [23].

Based on the *C* acquired, we can calculate *δ* from Equation (4), and then *I_ref_* from Equation (2) reversely. The red curve in Figure 4(a) is the calculated relationship between the pressure applied and reflection intensity. Obviously, it matches the experimental result very well.

From Equations (2) and (3), both reflected and transmitted light intensities are dependent on the input power. Any variation in the optical power would inevitably cause undesirable signal variation, affecting the precision and stability of the sensor. In traditional polarimetric fiber sensors, a special two-detector system is used to avoid this problem [24,25]. A free-space beam splitter (e.g., Wollaston Prism) is introduced into the configuration, which splits the linearly polarized light beam into two beams polarized normal to each other. With appropriate detection and data processing, this problem can be solved. However, the all-fiber light path is broken, and the need to use additional optical components brings about packaging difficulties that restrict the reliability of these fiber sensors.

However, in our system, if we record both reflection and transmission output change synchronously, after data procession:

(6) $$\frac{I_{\mathrm{trans}}-I_{\mathrm{ref}}}{I_{\mathrm{trans}}+I_{\mathrm{ref}}}=\text{cos}\delta$$

The result is independent of input light intensity while it is just affected by the induced phase retardation. In this convenient way, we are able to significantly reduce system fluctuations owing to unstable power source.

**Figure 5.**
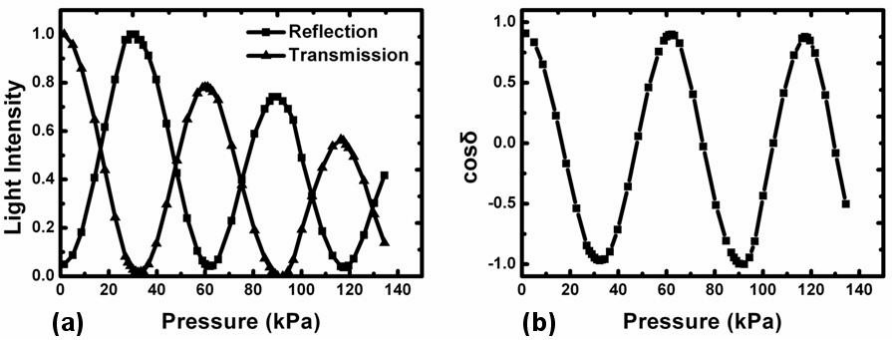
**(a)** The pressure induced reflection and transmission change with reducing input light intensity; **(b)** The pressure induced phase retardation after data procession.

To prove this idea, we set the variable attenuator to decrease by 0.03 dB every 15 seconds, beginning from −1.495 dB to mimic the light source instability. With other conditions unchanged, we repeat the pressure sensing experiment mentioned above. Figure 5(a) illustrates the result: both reflection and transmission output intensity decreases evidently due to reduced input intensity.

Figure 5(b) shows the result after data procession according to Equation (6). Obviously, input light intensity decrease does not have any effect on this curve. The induced phase retardation could be calculated easily. Hence, taking advantage of the trait of its structure, our pressure sensor successfully suppresses the influence of unstable source and unpredictable propagation fluctuation, while preserving the integrity of the all-fiber optical path, which may improve the sensor’s performance and reliability.

Our pressure sensing is demonstrated in Figures 4 and 5. In fact, the NWGFP still has other applications as well. Besides 1550 nm, it can also work in other IR wavelengths, only if the wavelength is far larger than its period. What’s more, principally, it may detect any parameter changes associated with light polarization inside a fiber, e.g., environmental magnetic field and electric current in surrounding wires. These experiments are already under way in our laboratory.

## 4. Conclusions

We have elaborated a fiber polarizer and pressure sensor based on a nano-wire grid. Although its polarizing performance is not ideal, it is still qualified as a pressure sensor. Without introducing any free-space component, it can also avoid the influence of source fluctuation and propagation loss with ease, which makes a prominent merit of our sensor. This novel feature gives rise to its practical use in the sensing field.
